# The Mechanism of Mitochondrial Injury in Alpha-1 Antitrypsin Deficiency Mediated Liver Disease

**DOI:** 10.3390/ijms222413255

**Published:** 2021-12-09

**Authors:** Nazli Khodayari, Rejean L. Wang, Regina Oshins, Yuanqing Lu, Michael Millett, Alek M. Aranyos, Sayedamin Mostofizadeh, Yogesh Scindia, Tammy O. Flagg, Mark Brantly

**Affiliations:** 1 Division of Pulmonary, Department of Medicine, University of Florida, Gainesville, FL 32610, USA; sunshinebear008@yahoo.com (R.L.W.); Regina.Oshins@medicine.ufl.edu (R.O.); yuanqing.lu@medicine.ufl.edu (Y.L.); michaelmillett@ufl.edu (M.M.); Alek.Aranyos@medicine.ufl.edu (A.M.A.); yogesh.scindia@medicine.ufl.edu (Y.S.); Tammy.Flagg@medicine.ufl.edu (T.O.F.); 2 Department of Pathology, Immunology and Laboratory Medicine, University of Florida, Gainesville, FL 32610, USA; s.mostofizadeh@ufl.edu

**Keywords:** alpha-1 antitrypsin deficiency, protein misfolding, lipid droplets, mitochondrial injury

## Abstract

Alpha-1 antitrypsin deficiency (AATD) is caused by a single mutation in the SERPINA1 gene, which culminates in the accumulation of misfolded alpha-1 antitrypsin (ZAAT) within the endoplasmic reticulum (ER) of hepatocytes. AATD is associated with liver disease resulting from hepatocyte injury due to ZAAT-mediated toxic gain-of-function and ER stress. There is evidence of mitochondrial damage in AATD-mediated liver disease; however, the mechanism by which hepatocyte retention of aggregated ZAAT leads to mitochondrial injury is unknown. Previous studies have shown that ER stress is associated with both high concentrations of fatty acids and mitochondrial dysfunction in hepatocytes. Using a human AAT transgenic mouse model and hepatocyte cell lines, we show abnormal mitochondrial morphology and function, and dysregulated lipid metabolism, which are associated with hepatic expression and accumulation of ZAAT. We also describe a novel mechanism of ZAAT-mediated mitochondrial dysfunction. We provide evidence that misfolded ZAAT translocates to the mitochondria for degradation. Furthermore, inhibition of ZAAT expression restores the mitochondrial function in ZAAT-expressing hepatocytes. Altogether, our results show that ZAAT aggregation in hepatocytes leads to mitochondrial dysfunction. Our findings suggest a plausible model for AATD liver injury and the possibility of mechanism-based therapeutic interventions for AATD liver disease.

## 1. Introduction

Alpha-1 antitrypsin (AAT), the most abundant serine protease inhibitor in plasma, is primarily produced in the liver [1]. AAT is secreted into circulation by hepatocytes and contributes to innate immunity by inhibiting destructive neutrophil proteases, including elastase, cathepsin G, and proteinase 3 [2]. Alpha-1 antitrypsin deficiency (AATD) is most often caused by the Z mutant variant of AAT (ZAAT), a deficiency allele of the SERPINA1 gene [3], resulting in misfolding, aggregation, and accumulation of ZAAT aggregates followed by endoplasmic reticulum (ER) stress in the hepatocytes [4,5]. Proteotoxic consequences of the accumulation of ZAAT in the hepatocytes predisposes affected individuals to AATD-mediated liver disease, starting with acute injury and progressing to fibrosis, cirrhosis, and hepatocellular carcinoma [6]. Resulting low levels of circulating AAT leads to uncontrolled proteolytic activity and induction of inflammatory processes in different tissues, especially lungs [7]. Several studies show that intrahepatic accumulation of periodic acid-Schiff-positive, diastase-resistant (PASD) globules representing aggregated ZAAT are associated with liver injury and hepatocellular carcinoma in AATD transgenic mouse models [8]. Together, these breakthroughs have shown that proteotoxicity of ZAAT accumulation in hepatocytes is the dominant mechanism for liver disease mediated by AATD. Despite this, there is limited knowledge about mechanisms that underlie the gain of toxicity associated with ZAAT and resultant hepatocellular injury.

Intracellular misfolded protein aggregates play a critical role in the pathogenesis of a wide range of diseases by altering key functions of a variety of organelles, such as ER, endosomes, lysosomes, the proteasome degradation system, and mitochondria [9]. Mitochondria, intracellular double membrane-bound structures, are highly dynamic organelles in which respiration and energy generation occur [10]. Hepatocytes contain a high density of mitochondria due to the central role of the liver in carbohydrate, lipid, and protein metabolism homeostasis [11]. Unique features of hepatic mitochondria as integration sites of metabolism require their correct functioning to maintain liver homeostasis and prevent liver disease [12]. Mitochondrial dysfunction associated with chronic liver diseases most often presents as damaged mitochondria with depleted glutathione (GSH) and respiratory complex alterations that produce and release abnormal reactive oxygen species (ROS) [12]. Mitochondrial injury in AATD-mediated liver disease has been previously shown by abnormal mitochondrial morphology and altered mitochondrial autophagy in the liver of AATD individuals and transgenic AATD mouse models [11]. It is clear that mitochondrial dysfunction alone is sufficient to trigger cell death in primary mitochondrial diseases resulting from mutations in mitochondrial DNA or nuclear DNA encoding of mitochondrial proteins [13]. However, it is less clear whether mitochondrial dysfunction seen in AATD-mediated liver disease is necessary for pathogenesis of the disease that has ER stress caused by hepatic accumulation of misfolded ZAAT as its most prominent feature.

ER stress is associated with lipid droplet formation. Accumulation of lipid droplets has been observed as a general downstream effect of ER stress [14] in many protein-misfolding diseases [15,16,17] such as AATD [18]. Lipid droplets are cytoplasmic organelles that serve as storage compartments for lipids in animal cells [19]. Previous studies have shown that in cells with high fatty acid storage and oxidation capacity, lipid droplets also associate with mitochondria. Notably, lipid droplets have been shown to regulate mitochondrial dynamics by ensuring maximum oxidative metabolism and homeostasis [20]. Mitochondrial recruitment to the surface of lipid droplets mediated by perilipin 5 (Plin5), a surface protein of lipid droplets, leads to bioenergetic changes in mitochondria [21]. In addition to lipid droplets, mitochondria are also associated with the ER. Recent studies support that ER stress affects mitochondria by allowing aggregated misfolded proteins to be translocated to them [22]. While mechanistic details of this process require further investigation, it is also possible that accumulation of misfolded proteins inside mitochondria overwhelms mitochondrial proteostasis or disrupts functional activities of the mitochondria, leading to organelle dysfunction [22].

In the present study, we investigated the morphology, bioenergetic activity, and localization of the mitochondria in AATD-mediated liver disease using a human AAT transgenic mouse model and an AATD cell culture model. We show that ZAAT accumulation in hepatocytes is associated with hepatic alteration of lipid metabolism pathways and accumulation of lipid droplets bound to mitochondria. Our results demonstrate the import and accumulation of misfolded ZAAT aggregates in mitochondria, which is accompanied by altered morphology and distinct bioenergetics of hepatic mitochondria in AATD. Mitochondrial targeting of ZAAT in vivo and in vitro and mitochondrial dysfunction in ZAAT-expressing hepatocytes suggest that mitochondrial localization of misfolded ZAAT may be the cause of mitochondrial dysfunction, as seen in the pathogenesis of AATD-mediated liver disease. These findings provide a novel specific mechanism to explain how misfolded ZAAT directly impairs mitochondrial function and causes toxic gain-of-function in hepatocytes.

## 2. Results

### 2.1. Hepatic Accumulation of Human ZAAT in Pi*Z Transgenic Mice Leads to Lipid Accumulation in the Liver of Transgenic Mice

**Figure 1:**
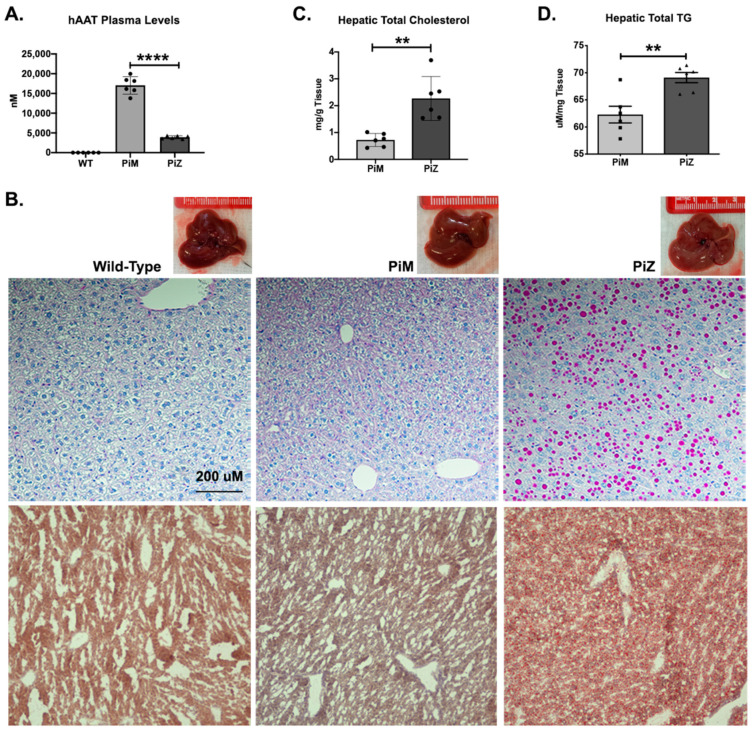
Characterization of Pi*M and PI*Z transgenic mice. (**A**) Enzyme-linked immunosorbent assay (ELISA) was used to measure human alpha-1 antitrypsin (hAAT) from the plasma of wild-type, Pi*M, and Pi*Z transgenic mice (*n* = 6); (**B**) PAS-D staining using immunohistochemistry on liver tissue of wild-type, Pi*M, and Pi*Z mice (top row). Neutral lipids and lipid droplets were assessed in Pi*Z transgenic mice and compared to Pi*M and wild type using H&E and Oil Red O staining, respectively (middle and bottom rows). (**C**) Hepatic total cholesterol and (**D**) triglycerides (TG) quantified by colorimetric assay from the Pi*M and Pi*Z mice liver tissue. ** *p* < 0.001, **** *p* < 0.0001.

Our transgenic human AAT (hAAT) mouse model for the investigation of AATD liver disease exhibited AAT expression patterns resembling those of humans. As observed in AATD individuals with liver disease, significant ZAAT aggregation was observed in human Z mutant AAT transgenic mouse (Pi*Z) livers and could be visualized with periodic acid–Schiff staining after diastase treatment (PAS-D). These PAS-D–positive globules accumulated in the ER and caused ER stress, resulting in hepatic injury in Pi*Z mice compared to human normal AAT transgenic mice (Pi*M) and wild-type animals [23]. In our mouse model of AATD, the evaluation of plasma liver enzyme levels showed no significant differences between Pi*M and Pi*Z transgenic mice, similar to humans [24] (data not shown). In addition to a significant reduction in hAAT secreted to the plasma (Figure 1A), our data from the evaluation of the livers from transgenic mice showed that the livers of Pi*Z transgenic mice appeared paler than the controls (Figure 1B). The presence of ZAAT aggregates in the liver of Pi*Z transgenic mice was evaluated using PAS-D staining (top row). The results showed the presence of PAS-D globules in the liver of Pi*Z transgenic mice starting at 3 months old (data not shown) and the mice showed a spike in liver PAS-D globules when they were approximately 6 months old (Figure 1B). Oil Red O staining of the liver tissue also revealed that there were increased lipid droplets in the livers of Pi*Z transgenic mice compared to Pi*M and wild-type mice (bottom row), demonstrating a defect in lipid metabolism (Figure 1B). Our results also indicated the induction of hepatic steatosis, quantified by measuring total cholesterol and triglyceride (TG) levels extracted from liver tissues of wild-type and transgenic mice (Figure 1C,D).

### 2.2. Transcriptome Analyses Suggest Pi*Z Transgenic Mice Acquire Mitochondrial Abnormalities and Changes in Lipid Metabolism Pathways

Given the abnormalities in protein trafficking observed in the ER of hepatocytes expressing ZAAT [25] and the accumulation of human ZAAT in the liver of Pi*Z transgenic mice compared to wild-type and Pi*M transgenic mice (Figure S1), we hypothesized that ZAAT accumulation in the ER of hepatocytes could be associated with alterations in specific pathways related to stress responses.

**Figure 2:**
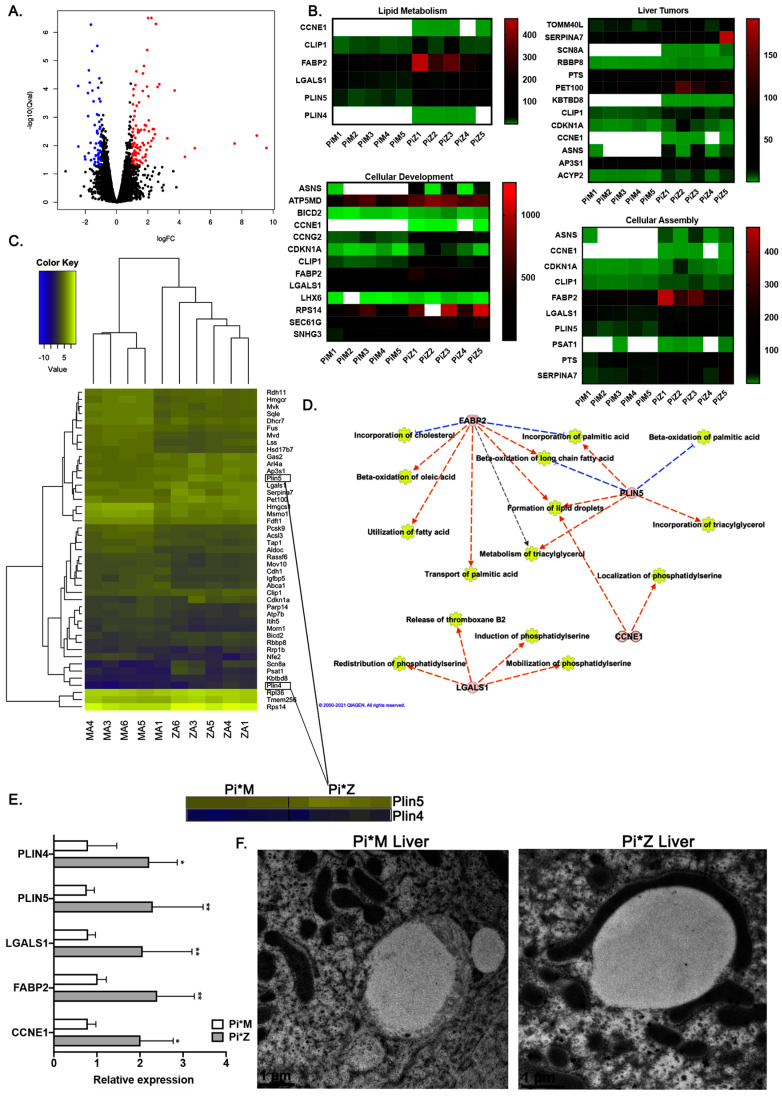
Dysregulation of lipid metabolism pathways and mitochondrial abnormalities in Pi*Z transgenic mice. (**A**) Volcano plot of log2 fold change (logFC) vs. *p*-value between Pi*Z and Pi*M liver tissues. Black circles represent RNAs with a *p* > 0.05, blue circles represent downregulated RNAs with a *p* < 0.05 and a fold change greater than 2, and red circles represent upregulated RNAs with a *p* < 0.05 and a fold change greater than 2. (**B**) The related heatmap of the enriched pathways identified by IPA (ingenuity pathway analysis) from differentially expressed RNAs between Pi*M and Pi*Z mice liver tissues. (**C**) The related heatmap of the differentially expressed RNAs between Pi*M and Pi*Z mice liver tissues involved in lipid metabolism pathway. (**D**) Functional repertoire of 4 upregulated RNAs in Pi*Z transgenic mice liver tissues involved in the elevation of lipid metabolism pathway using IPA software. Blue lines indicate direct inhibition and orange lines indicate direct activation. (**E**) Relative expression of Ccne1, Fabp2, Lgals1, Plin5, and Plin4 genes using qRT-PCR data derived from Pi*M and Pi*Z transgenic mice liver tissues. (**F**) Immuno-electron microscopy results determining the morphology and localization of the mitochondria in the liver tissue from Pi*M and Pi*Z transgenic mice. * *p* < 0.05, ** *p* < 0.001.

To investigate this hypothesis and understand the implications of ZAAT accumulation in hepatocytes, RNA sequencing (RNA-seq) was performed on the liver tissues isolated from age- and sex-matched Pi*Z and Pi*M transgenic mice (*n* = 5). Statistical analysis revealed 156 genes with significantly different expression (at the thresholds of false-discovery rate (FDR) = 0.05 and fold change > 2), the majority of which were protein-coding genes (Figure 2A).

Of the 156 differentially expressed genes, 96 were upregulated in Pi*Z liver tissues, whereas 60 were downregulated. Ingenuity pathway analysis (Qiagen IPA, Germantown, MD, USA) of the RNA-Seq data was employed to identify the key pathways changed in response to ZAAT accumulation in the mouse liver tissue. Lipid metabolism was one of the top-ranked enriched pathways in Pi*Z liver tissues (FDR  <  0.05) (Figure 2B). Pathways related to the liver tumors, cellular development, and assembly were also enriched in Pi*Z liver tissues (Figure 2B). Using ingenuity pathway analysis, we observed a total of 50 differentially expressed genes in Pi*Z liver tissues that have a significant role in lipid metabolism pathways. We also found five upregulated genes, including FABP2, PLIN5, PLIN4, CCNE1, and LGALS1, which are associated with overactivation of lipid metabolism pathways (Figure 2C–E). Additional analysis of the RNA-Seq dataset and qPCR validation revealed that there was a robust increase in the expression of FABP2, PLIN5, PLIN4, CCNE1, and LGALS1 genes in Pi*Z liver tissues compared to Pi*M (Figure 2E). A previous study [26] showed that Plin5 promoted lipid droplet contact with mitochondria, and subsequently we validated this phenotype in Pi*Z mouse liver tissue. Results from electron microscopy analysis of Pi*Z and Pi*M liver tissue confirmed that the presence of abnormal elongated mitochondria with defused membranes in Pi*Z mouse liver tissue was highly associated with lipid droplets (Figure 2F).

### 2.3. Hepatic Human ZAAT Accumulation Is Associated with Mitochondrial Dysfunction in the Liver of Transgenic Mice

**Figure 3:**
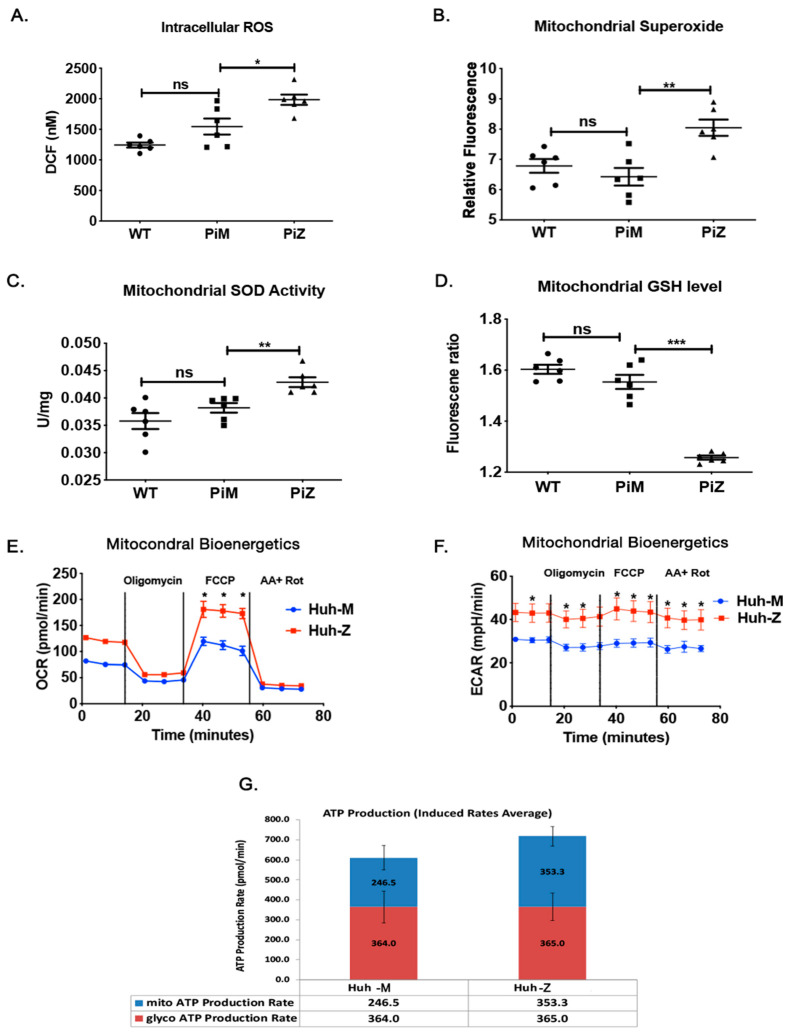
ZAAT-induced hepatic mitochondrial dysfunction. (**A**) Hepatic intracellular ROS levels in wild-type, Pi*M, and Pi*Z mice by measuring the DCF fluorescent signal in fresh liver sections. (**B**) Hepatic mitochondrial superoxide levels in wild-type, Pi*M, and Pi*Z mice. (**C**) Activity of hepatic mitochondrial superoxide dismutase (SOD) enzyme measured by superoxide dismutase assay kit in wild-type, Pi*M, and Pi*Z mice. (**D**) Hepatic mitochondrial glutathione (GSH) levels of wild-type, Pi*M, and Pi*Z mice. (**E**,**F**) The OCR (oxygen consumption rate) and ECAR (extracellular acidification rate) in control and ZAAT-expressing Huh7.5 cells measured by Seahorse assay followed by (**G**) the ATP production rate. ^ns^ nonsignificant, * *p* < 0.05, ** *p* < 0.001, *** *p* < 0.0001.

Hepatocytes of AATD mouse models [27] generate excessive reactive oxygen species (ROS), though their origin has not yet been investigated. Therefore, we stained fresh liver sections and confirmed the phenotype of our human AAT transgenic mice by measuring intracellular ROS in wild-type, Pi*M, and Pi*Z transgenic mice with DCF (full form) as an indicator of ROS. Our results show that compared to Pi*M and wild-type mouse liver tissue, ROS levels were significantly higher in the liver tissue of Pi*Z mice (Figure 3A). As was already observed by other groups [27], our report confirms the contribution of ROS to AATD-mediated liver injury. To identify the source of ROS, we analyzed the mitochondria from the livers of these animals. Compared to wild-type and Pi*M, the mitochondrial superoxide levels (Figure 3B) were significantly elevated in Pi*Z transgenic mice. An increase in mitochondrial superoxide was associated with a concomitant increase in mitochondrial superoxide dismutase (SOD) in the same cohort of mice (Figure 3C). Furthermore, the endogenous anti-oxidant, reduced glutathione (GSH) levels were significantly lower in the mitochondria of the Pi*Z mutants (Figure 3D), which agreed well with the increase in mitochondrial ROS.

To test whether the increase in mitochondrial ROS in the Pi*Z transgenic mice liver tissue couples with ATP production, we measured ATP production in Huh7.5 expressing MAAT or ZAAT. As expected, increased ZAAT-expressing cells produced more ATP than MAAT-expressing cells. To confirm that increased ATP production in ZAAT-expressing cells is due to an increase cellular metabolic rate, we measured oxygen consumption rate (OCR), which corresponds to OXPHOS, and extracellular acidification rate (ECAR), which is primarily attributed to glycolysis. Indeed, the ZAAT-expressing Huh7.5 cells (comparable to Pi*Z) increased OCR (Figure 3E) and ECAR (Figure 3F) more than MAAT-expressing Huh7.5 cells (comparable to Pi*M) did. Collectively, these results indicate that Pi*Z or ZAAT expression and accumulation is associated with an increase in ROS and ATP production, which is a result of increased mitochondrial bioenergetics. This finding is in line with the previous report indicating the contribution of ROS to AATD-mediated liver injury [27].

### 2.4. Hepatic Human ZAAT Accumulation Impairs Mitochondrial Membrane Integrity within the Liver of Transgenic Mice

**Figure 4:**
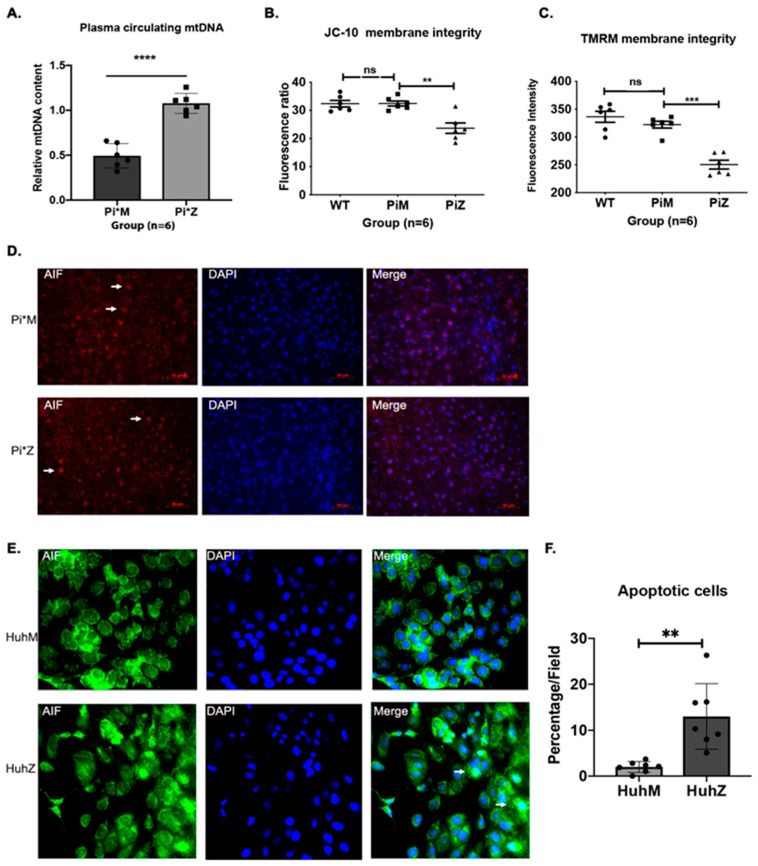
ZAAT-mediated caspase independent apoptosis in hepatocytes. (**A**) Relative mtDNA levels in the plasma of Pi*M and Pi*Z mice, quantified by RT-PCR. (**B**) Mitochondrial membrane integrity measured by JC-10 fluorescence assay in wild-type, Pi*M, and Pi*Z liver homogenates. (**C**) ΔψΜ levels determined by monitoring fluorescence of tetra-methyl-rhodamine methyl ester (TMRM). (**D**) Immunostaining for endogenous AIF (apoptosis-inducing factor) in Pi*M and Pi*Z mice liver tissue and (**E**,**F**) in MAAT- and ZAAT-expressing Huh7.5 cells. Arrows show the cytoplasmic localization of AIF in Pi*M mouse liver tissue and nuclear localization of AIF in Pi*Z mouse liver tissue. The bar chart shows the percentage of apoptotic cells in each field of MAAT- and ZAAT-expressing Huh7.5 cells based on nuclear localization of AIF. ^ns^ nonsignificant, ** *p* < 0.001, *** *p* < 0.0001, **** *p* < 0.00001.

Circulating mitochondrial DNA (mtDNA) can be released from apoptotic and necrotic cells, reflecting cellular and tissue injury due to mitochondrial damage [28]. Therefore, plasma-circulating mtDNA levels were investigated in Pi*M and Pi*Z mice (*n* = 6) and quantified by RT-PCR [29]. Results show that the circulating mtDNA copy number was significantly increased in the Pi*Z mice (Figure 4A). Next, we evaluated mitochondrial membrane integrity by measuring mitochondrial membrane potential in the liver of Pi*M and Pi*Z transgenic mice. Mitochondrial membrane potential is an indicator of healthy cells and ΔψΜ is an important parameter of mitochondrial function and mitochondria-mediated apoptosis [30]. Thus, ΔψΜ was estimated using JC-10 (a lipophilic cationic dye) necrotic and apoptotic cells because of monomeric JC-10 diffusion (green fluorescence).

We observed a reduction in JC-10 fluorescence at excitation/emission wavelengths of 490/525 nm and 490/590 nm in Pi*Z liver homogenates compared to Pi*M and wild type (Figure 4B). Similarly, Pi*Z liver homogenates showed lower ΔψΜ by monitoring the fluorescence of tetra-methyl-rhodamine methyl ester (TMRM), a cationic lipid-soluble probe that accumulates in energized mitochondria (Figure 4C). Permeabilization of mitochondria increases the mitochondrial release of apoptosis-inducing factor (AIF) to the cytoplasm, which plays a central role in caspase-independent cell death [31]. Released mitochondrial AIF is translocated to the nucleus following an apoptotic stimulus, inducing chromatin condensation in a caspase-independent manner [32]. Therefore, we investigated whether AIF is translocated to the nucleus during mitochondrial permeabilization of Pi*Z hepatic mitochondria. Immunostaining analysis exhibited nuclear translocation of endogenous AIF in Pi*Z liver tissues, but cytoplasmic localization in Pi*M liver tissues (Figure 4D). A similar trend was observed in ZAAT-expressing Huh7.5 cells compared to MAAT-expressing Huh7.5 cells (Figure 4E,F).

### 2.5. AAT Protein Associates with Mitochondria In Vitro and In Vivo

**Figure 5:**
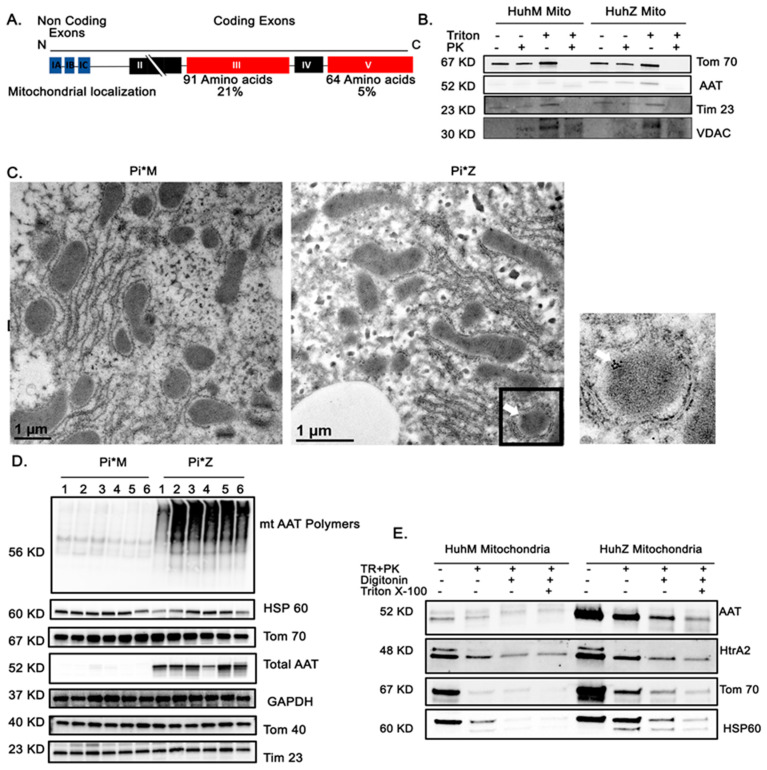
AAT association with hepatocyte mitochondria. (**A**) Alignment of the sequences of amino acids in the AAT molecule. (**B**) Western blot analysis showing sequential Triton and proteinase K treatments of the mitochondrial fraction of MAAT- and ZAAT-expressing Huh7.5 cells. A voltage-dependent anion-selective channel (VDAC) was loaded as mitochondrial loading control. (**C**) Immuno-electron microscopy results determining the morphology and localization of the mitochondria in the liver tissue from Pi*M and Pi*Z transgenic mice. Immunogold-labeled antibody against hAAT was used to detect mitochondrial hAAT (white arrow). ER entrapped between lipid droplets and mitochondria is indicated by the black arrow. (**D**) Native, non-denaturing Western blot analysis of hepatic mitochondrial ZAAT aggregates in the 6 Pi*M (1–6) and 6 Pi*Z (1–6) mouse liver tissue as well as the total levels of AAT, mitochondrial markers, and GAPDH as loading control. (**E**) Western blot analysis of isolated mitochondrial fraction of Huh7.5 cells, showing the exact localization of AAT in permeabilized mitochondria using proteinase K, digitonin, and Triton X-100.

Mitochondrial targeting signals are rich in basic amino acids at their N-terminus, though some proteins have these signals at the C-terminus or internally [33]. AAT is a 394 amino acid soluble protein containing four coding exons [34]. Alignment of exons 3 and 5 revealed basic amino acids in the AAT molecule, suggesting the internal section, and with less probability the C-terminus, resemble properties of mitochondrial targeting sequences. We used WoLFSORT to predict signal-targeting activities. Multi-subcellular localization of AAT, including 21% mitochondrial localization when the exon 3 sequence was analyzed and 5% mitochondrial localization when the exon 5 sequence was used, suggests that the internal section may function as a mitochondrial targeting signal (Figure 5A). It has been shown that misfolded protein aggregates bind to mitochondria via interaction with import receptors such as Tom70, facilitating the import and degradation of misfolded proteins by the mitochondrial proteasome system [22]. To determine whether ZAAT is directly linked to hepatic mitochondria in our model, we performed a purification of mitochondria and Western blot analysis. Sequential trypsin and proteinase K (PK) treatments were used to eliminate surface AAT aggregates from mitochondria. In a mitochondrial pellet purified from ZAAT-expressing Huh7.5 cells, a fraction of ZAAT that was protected from trypsin was detected (Figure S2A). The fraction of MAAT protected from PK was low in the purified mitochondrial pellet of MAAT-expressing Huh7.5 cells (Figure 5B). We also used purified mitochondria from monocyte-derived macrophages and showed that mitochondrial localization of AAT is similar to that in hepatocytes (Figure S2B). This suggests that localization of AAT in mitochondria is a common characteristic of AAT-expressing cells and not just hepatocytes. To confirm this novel observation, a double-immunofluorescence technique was used to investigate co-localization of AAT with Tom40, a mitochondrial resident protein. As Tom40 antibody gives diffuse immunostaining at lower magnification, they will overlap, regardless of physical co-localization; sections were examined using an oil lens (100X). In the merged picture, immunofluorescence of ZAAT was overlapped with Tom40 (Figure S2C). To subcellularly locate AAT in Pi*M and Pi*Z mouse liver tissues, we used immuno-electron microscopy and immunogold labeled AAT antibody. As shown in Figure 5C, gold particles were seen in the ER of hepatocytes in both Pi*M and Pi*Z mouse liver tissues at different densities. The density of gold particles was relatively low in the ER of Pi*M mouse liver tissues, whereas dense gold particles were gathered in the ER of Pi*Z mouse liver tissues. In Pi*Z liver tissues, gold particles were also clustered inside hepatic mitochondria, whereas few were seen inside Pi*M hepatic mitochondria. (Figure 5C). Next, we investigated aggregation of mitochondrial ZAAT in liver tissues harvested from Pi*M and Pi*Z transgenic mice by detergent extraction and PK treatment. Native, non-denaturing Western blotting revealed that the majority of mitochondrial ZAAT was aggregated. Total levels of AAT, mitochondrial markers, and GAPDH were also detected in Pi*M and Pi*Z mouse liver tissue (*n* = 6) (Figure 5D). To investigate localization of AAT, mitochondrial outer and inner membranes were permeabilized with digitonin and Triton X-100, respectively [22], and subjected to Western blot. Total surface AAT aggregates were eliminated using proteinase K treatment. Immunoblots for AAT and markers of mitochondrial outer membrane (Tom70), inner membrane space (HtrA2), and matrix (Hsp60) showed that ZAAT was present in both the inner membrane space and the matrix (Figure 5E).

### 2.6. AAT Imports to the Mitochondria Using Hepatic Mitochondrial Import Machinery

**Figure 6:**
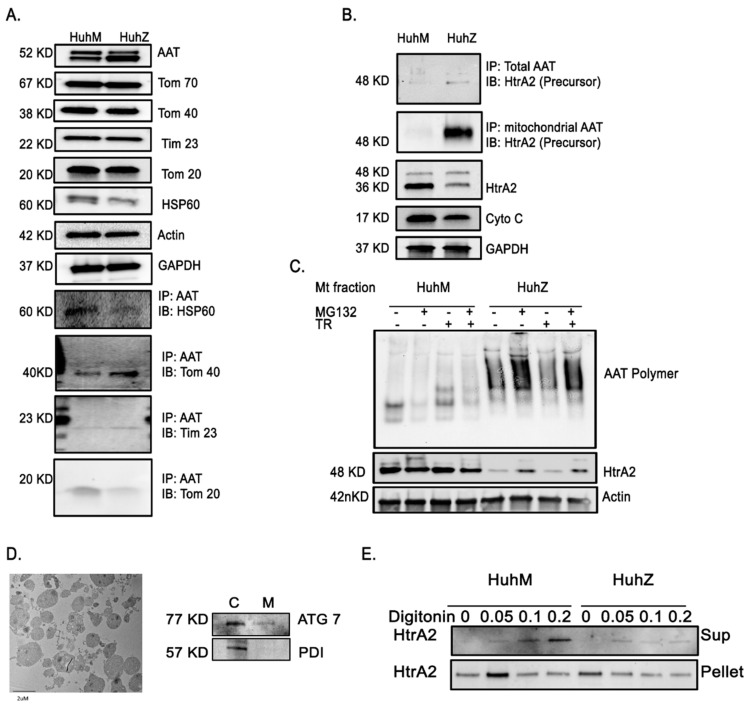
Mitochondrial interaction of AAT and HtrA2. (**A**) Detection of AAT translocation to the mitochondria through mitochondrial import machinery using co-immunoprecipitation analysis from total cell lysate, showing both MAAT and ZAAT interaction with Tom40, Tom20, and Tim23 in Huh7.5 cells. (**B**) Co-immunoprecipitation analysis from total Huh7.5 cell lysate, indicating mitochondrial ZAAT interaction with mitochondrial HtrA2. ZAAT interacts with the full-length precursor form of HtrA2. (**C**) The aggregation status of mitochondrial AAT and HtrA2 in the mitochondrial fraction of MAAT- and ZAAT-expressing Huh7.5 cells using proteasome inhibitor MG132 and trypsin. Actin was loaded as loading control. (**D**) Electron microscopy showing mitochondrial fraction purity and Western blot analysis of peroxisomal ATG7 and ER-associated chaperone PDI in cytoplasmic (C) and mitochondrial fraction (M), respectively. (**E**) Western blot analysis showing the mitochondrial release of HtrA2 in presence of different concentrations of digitonin form MAAT- and ZAAT-expressing hepatocytes.

An essential point to clarify regarding the effect of ZAAT on mitochondrial physiology is how ZAAT reaches mitochondria. The nature of targeting signals and interaction with mitochondrial receptors of AAT is important in understanding its role in mitochondrial dysfunction. Tom70, Tom20, and Tom22, mitochondrial translocases on the outer membrane, recognize mitochondrial import signals. Following recognition of signals by these receptors, proteins are transported through Tom40. Then, proteins are recognized by inner membrane translocases Tim22 and Tim23 [35]. We performed co-immunoprecipitation analysis of MAAT- and ZAAT-expressing Huh7.5 cells and observed that both MAAT and ZAAT bound to Tom40, Tom20, and Tim23 in Huh7.5 cells (Figure 6A), supporting the notion of AAT mitochondrial import through mitochondrial import machinery. No interaction between AAT and Tom70 or Tom22 was found. Further co-IP analysis revealed that mitochondrial ZAAT interacted with mitochondrial HtrA2 (Figure 6B). We also found that HtrA2 was downregulated in Huh7.5 cells expressing ZAAT, although no changes were detected in the levels of cytochrome C between Huh cells expressing M or ZAAT (Figure 6B). In the whole cell lysate, HtrA2 was detected as two main bands and ZAAT preferentially interacted with the full-length precursor form of HtrA2 (Figure 6B). To further investigate whether HtrA2 gets degraded as a part of ZAAT aggregates, we used 20 nM MG132 to inhibit the proteasome degradation machinery upon a 6 h incubation time, then we investigated the levels of ZAAT polymers within the mitochondria. We show that inhibition of the proteasomal degradation system using MG132 resulted in an increase in mitochondrial ZAAT polymers (Figure 6C), indicating that inhibition of ZAAT degradation leads to an increase in the mitochondrial import of ZAAT. Surprisingly, we observed that there was an increase in mitochondrial HtrA2 levels in the presence of MG132. These data indicate that low levels of HtrA2 in ZAAT-expressing hepatocytes, as well as Pi*Z mouse liver tissue (Figure S3A), may be due to HtrA2 proteasomal degradation as a consequence of forming a complex with ZAAT that has been tagged for degradation. To examine the behavior of mitochondrial HtrA2 in MAAT- and ZAAT-expressing hepatocytes, we used digitonin in increasing concentrations to permeabilize the outer and inner membranes of isolated mitochondria [36]. Digitonin is effective in solubilizing the sterol-rich mitochondrial outer membrane (and plasma membrane), but only at higher concentrations does it effectively solubilize the sterol-poor mitochondrial inner membrane. Mitochondrial fraction purity was confirmed by electron microscopy and Western blot for peroxisomal ATG7 and ER-associated chaperone PDI (Figure 6D). Digitonin was added at 0.05%, 0.1%, or 0.2% *v*/*v* to mitochondrial pellets in PBS, and supernatants were analyzed for the release of HtrA2. At 0.05% digitonin, HtrA2 partly dissociated from the mitochondrial pellet into the supernatant (Figure 6E). HtrA2 did not dissociate from digitonin-permeabilized mitochondria equally in MAAT- and ZAAT-expressing cells. At higher digitonin concentrations, mitochondrial HtrA2 release was not gradually increased in ZAAT-expressing Huh7.5 cells, indicating lower levels of HtrA2 release from permeabilized ZAAT mitochondria.

### 2.7. ZAAT Knockdown Reduces the Lipid Content of Hepatocytes and Restores Mitochondrial Function in ZAAT-Expressing Hepatocytes

**Figure 7:**
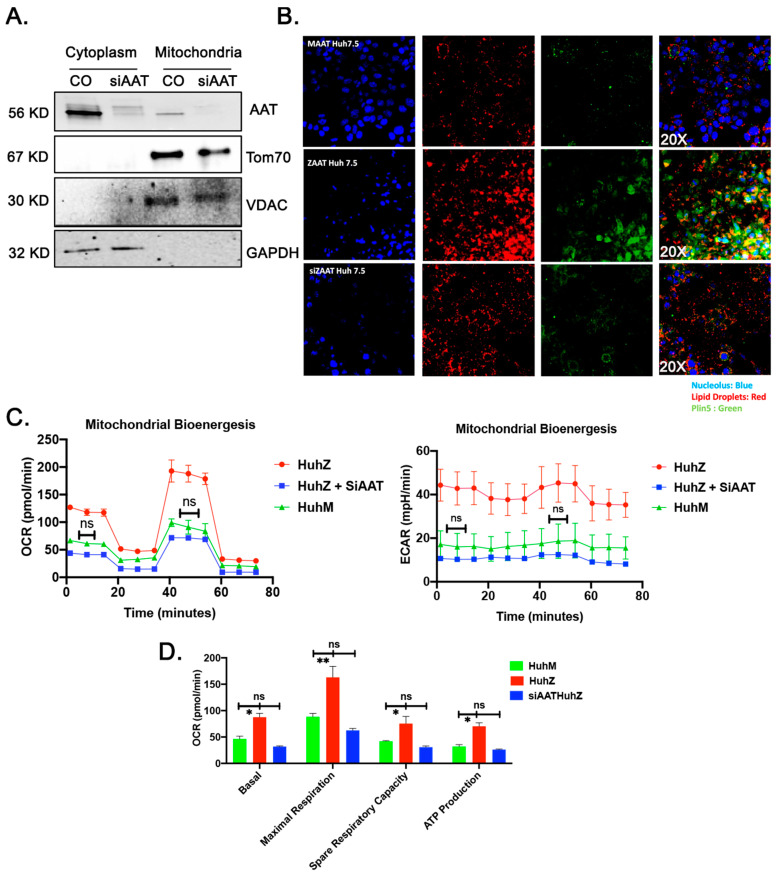
Restoration of the mitochondrial function in ZAAT knockdown hepatocytes. (**A**) Treatment with siAAT clears the ZAAT from the cytoplasm as well as mitochondrial fraction and downregulates the protein levels of Plin5. GAPDH, VDAC, and Tom70 were loaded as cytoplasmic and mitochondrial loading controls. (**B**) Immunofluorescence assay showing the reduction in lipid droplets (red) and Plin5 (green) in siAAT-treated Huh7.5 cells. (**C**,**D**) Seahorse experiment results showing reduced OCR, ECAR, and ATP production in siAAT-treated Huh7.5 cells compared to control ZAAT-expressing Huh7.5 cells and wild-type (MAAT) Huh7.5 cells. ^ns^ nonsignificant, * *p* < 0.05, ** *p* < 0.001.

To further confirm the pathogenic role of misfolded ZAAT in mitochondrial function, we treated ZAAT-expressing Huh7.5 cells with siRNA against AAT. As shown in Figure 7A compared to the si control group, treatment with siAAT cleared ZAAT aggregates from the cytoplasm as well as the mitochondrial fraction. We also observed downregulation of the protein levels of Plin5 in siAAT-treated hepatocytes and fewer lipid droplets compared to control ZAAT-expressing Huh7.5 cells (Figure 7B). Furthermore, siAAT-treated Huh7.5 cells showed reduced OCR, ECAR, and ATP production compared to control ZAAT-expressing Huh7.5 cells, comparable to wild-type (MAAT) Huh7.5 cells (Figure 7C,D). These results suggest that hepatic accumulation of misfolded ZAAT increases mitochondrial respiration associated with increased ROS generation, which can be resolved by elimination of the expression of misfolded ZAAT.

## 3. Discussion

AATD-mediated liver disease is associated with the retention of aberrantly folded ZAAT within the ER of hepatocytes, resulting in liver fibrosis, cirrhosis, and hepatocellular carcinoma via a toxic gain-of-function mechanism [37]. Previous studies have demonstrated abnormal hepatic mitochondrial morphology consistent with hepatocyte injury in AATD-mediated liver disease [2,11]. However, the underlying molecular mechanisms causing mitochondrial injury associated with AATD-mediated liver disease are poorly understood. Using a transgenic mouse model of AATD and a human cell line, our study describes a new mechanism for AATD-associated mitochondrial dysfunction by which hepatic accumulation of ZAAT is associated with hepatic expansion of lipid droplets, mitochondrial ZAAT accumulation, and high bioenergetic capacity. Of note, Benador et al. previously established the association of mitochondria with lipid droplets and showed that peri-droplet mitochondria have enhanced bioenergetic capacity [38]. Here, we elaborated on these observations by comparing liver tissue RNA-seq and proteomic datasets of human AAT transgenic mice to provide proof of functional connection between ZAAT accumulation, lipid droplet biogenesis, and mitochondrial abnormality. Furthermore, we discovered that AAT is transported to the hepatic mitochondria through mitochondrial import machinery. The accumulation of misfolded ZAAT inside hepatic mitochondria likely disrupts mitochondrial activities and overwhelms mitochondrial proteostasis, leading to organelle dysfunction followed by liver injury [39].

The ER plays a critical role in hepatic homeostasis, being the main synthesis site of both proteins and lipids in hepatocytes. Lipid droplets, as organelles associated with the ER, respond to disturbances in ER homeostasis so ER stress enhances the formation and accumulation of lipid droplets [14]. Emerging evidence has shown that lipid droplets act as protective mechanism against ER stress by rebalancing ER lipid homeostasis, removing misfolded proteins, and regulating autophagy [40,41]. Our in vivo data using wild-type and Z human AAT transgenic mice suggests that progressive accumulation of ZAAT globules within hepatocytes impairs lipid metabolism pathways and causes increased lipid content of the hepatocytes. This conclusion is further supported by hepatic transcriptomic data showing that lipid metabolism is the most significantly influenced metabolic process in Pi*Z mouse liver tissue.

It has been shown that lipid droplets recruit mitochondria as an adaptive response in physiological conditions, such as ER stress, that require lipid droplet expansion. Peri-droplet mitochondria are responsible for providing ATP for the ATP-demanding processes of lipid synthesis and cycling in response to ER stress [42]. For this purpose, mitochondria bound to lipid droplets are shown to express enhanced bioenergetic capacity that supports triacyl glyceride synthesis [38]. As expected, analysis of mitochondrial respiration in our in vivo and in vitro AATD model systems revealed higher oxygen consumption and energy production in ZAAT-expressing hepatocytes compared to wild-type controls, indicating continuously elevated mitochondrial activity. We found ZAAT-expressing hepatocytes have significantly higher OCR and ECAR levels, indicative of increased oxidative and glycolytic metabolism and a concomitant increase in ATP production compared to wild-type hepatocytes. It is well established that the Z-mutation leads to a proinflammatory phenotype and an increase in autophagy [43], which are energy-consuming processes. These processes can be sustained by increased mitochondrial metabolism and ATP production, as observed in ZAAT-expressing cells. Although we found that ZAAT-expressing cells have increased mitochondrial energetics, in vivo Pi*Z transgenic mouse liver tissue expressed higher mitochondrial ROS and lower mitochondrial GSH levels, which reinforces the ongoing hypothesis that accumulation of misfolded ZAAT contributes to hepatic ROS production [44]. Furthermore, the TEM observation from our transgenic Pi*Z mouse liver tissue offers an insight into the subcellular anatomy of how increased mitochondrial recruitment to lipid droplets occurs in AATD-mediated liver disease. Our data show a strong association of lipid droplets with the ER and mitochondria in Pi*Z mouse liver tissue, which is consistent with recent studies on the role of mitochondria in the metabolic reprogramming of cells by elongation of the mitochondrial network to sustain ATP production [45]. Even more intriguing is that our transcriptome analyses from Pi*M and Pi*Z mouse liver tissues identified a significantly upregulated gene called Plin5 in Pi*Z transgenic mouse liver tissue, which is known to have a strong regulatory role in the recruitment of mitochondria to lipid droplets [21]. Based on current knowledge, Plin5 is a scaffolding protein with tissue expression limited to oxidative tissues, such as heart and liver, facilitating lipid droplet–mitochondria cellular reorganization. Plin5 has been shown to transiently entrap bioactive lipids in lipid droplets close to mitochondria at times of increased cellular fatty acid influx [21]. Downregulation of Plin5 and reduced lipid contents follow ZAAT clearance in hepatocytes, suggests that Plin5 may play a role in protection against ER stress and cellular lipotoxicity in oxidative cells with high energy demands.

Here, we show that the presence of misfolded ZAAT within hepatocytes is associated with increased mitochondrial oxygen consumption and ATP production. This particular remodeling of mitochondrial metabolism leads to ROS production and mitochondrial permeability, which triggers specific cell-signaling pathways, resulting in oxidative and reductive stresses with the consequent onset of numerous pathologies or even cell and organismal death [46]. There is morphological evidence for mitophagy in the liver of AATD individuals and Pi*Z mice, which can be an indication of mitochondrial injury [11,47]. Mitochondria are also one of the main regulators of apoptosis, mainly through the permeabilization of the mitochondrial membrane and the release of proapoptotic proteins into the cytoplasm [48]. In this regard, Glasgow et al. reported that the ZAAT mutation is associated with decreased mitochondrial membrane potential. Previous studies also observed that ER stress caused by the accumulation of ZAAT induces apoptosis via caspase-12 activation in mouse liver tissues and caspase-4 in human hepatocytes [49]. Our findings indicate the dissipation of △*ψM* in ZAAT hepato-mitochondria compared to wild-type hepato-mitochondria, which is the primary change in mitochondrial integrity during apoptosis [48]. In agreement with this, liver tissue from Pi*Z mice as well as ZAAT-expressing Huh7.5 cells showed nuclear localization of AIF, which can initiate nuclear DNA fragmentation in apoptotic cells [50]. All together, these results support the conclusion that mitochondrial dysfunction induced by hepatic ZAAT accumulation results in a change in hepato-mitochondrial integrity and nuclear translocation of AIF to initiate apoptosis. Proliferation mediated by apoptosis, which restores tissue function during injuries, is beneficial for the organism, as it allows tissues to easily remove damaged cells and replace them with the progeny of healthy neighbor cells [51].

Hepatic aggregation and polymerization of misfolded ZAAT represents a key pathological feature of AATD-mediated liver disease. Much energy has been devoted to understanding pathways responsible for ZAAT degradation. Recently, a mitochondria-mediated degradation pathway was described that explains the import of aggregation-prone proteins into the mitochondria for degradation by mitochondrial proteases [22]. Based on this observation, it is reasonable to investigate whether mitochondrial dysfunction in AATD is associated with ZAAT aggregation at the mitochondrial level. Using bioinformatic software analysis, we characterized the putative mitochondrial targeting signal for the AAT protein. We found that exon 3 of the AAT protein contains 91 amino acids and evenly spaced positive residues, which is a critical characteristic of a mitochondrial targeting signal [52]. The Western blot analysis and immunoelectron microscopy data presented here provide convincing evidence that AAT is translocated to the mitochondria in AAT expressing cells, including hepatocytes and monocytes. Although we observed that cytoplasmic accumulation of both M and Z variants of the AAT protein may lead to mitochondrial import of both variants of AAT protein (Figure S2), our disease model shows that greater amounts of ZAAT are detectable in the hepatic mitochondrial fraction. We also observed that ZAAT aggregates in polymeric form within mitochondria. There are some possible reasons for greater mitochondrial import of ZAAT. One is that mitochondrial targeting of AAT is increased during pathogenesis of AATD. A few studies have addressed determinants of the cellular fate of misfolded ZAAT, including retention in the ER and disposal by ER degradative pathways. Seminal works [53] have shown how the Z mutation of AAT confers an unstable polymerogenic intermediate conformation in the ER of hepatocytes. Misfolded ZAAT translocated to the cytoplasm is degraded by the proteasome system and autophagy [11,47]. Notably, delayed degradation of misfolded ZAAT has been observed, which contributes to cytoplasmic accumulation of fully synthesized ZAAT [54]. The overwhelming majority of cytoplasmic ZAAT contains a putative mitochondrial targeting sequence, which, when accompanied by cytoplasmic Hsp70 (Figure S3B) and DNAJ chaperones [55], may interact with the TOM complex in the outer mitochondrial membrane [56]. Another possibility is that cytosolic misfolded ZAAT are imported into mitochondria for degradation. This pathway appears to be crucial for the turnover of aggregated proteins [22]. Mitochondrial misfolded proteins can be eliminated by different systems, including ubiquitin-dependent degradation and mitophagy [57]. Here, using proteasome inhibitor in ZAAT-expressing hepatocytes, we show an increase in the levels of mitochondrial ZAAT aggregates. However, the idea of the degradation of ZAAT aggregates by mitochondrial degradation machinery warrants further study.

If, as we hypothesize, hepatic ZAAT accumulation induces mitochondrial dysfunction, ZAAT silencing should alleviate mitochondrial dysfunction. Our experimental results show that suppression of ZAAT expression using siRNA against AAT results in the absence of ZAAT polymers within the mitochondria and restores the function of hepatic mitochondria in ZAAT-expressing Huh7.5 cells. This indicates that misfolded ZAAT has a negative effect on mitochondrial function in hepatocytes, which is in line with the positive correlation of ZAAT aggregates and liver injury in AATD individuals [58].

**Figure 8:**
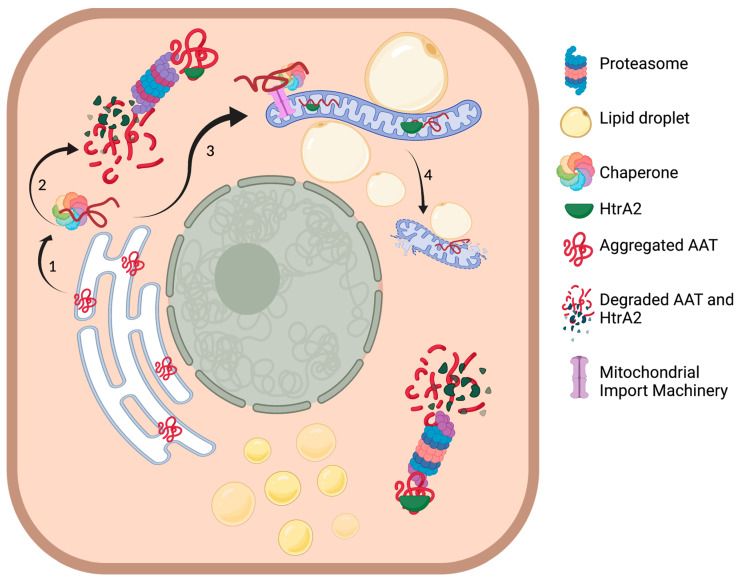
ZAAT-mediated mitochondrial injury within the hepatocytes. (1) Hsp70 binds to ZAAT to prevent aggregation of the mutated protein targeted for proteasomal degradation. (2) Proteasomal degradation of ZAAT. (3) Interaction of the cytoplasmic chaperone with mitochondrial import machinery leading to ZAAT mitochondrial translocation. (4) Disruption of mitochondrial membrane integrity and mitochondrial dysfunction. Figure was made by BioRender.

Collectively, the present study describes the physical contact of abnormal mitochondria with enhanced bioenergetic capacity and lipid droplets in ZAAT-expressing hepatocytes. Furthermore, our study highlights the mitochondrial import of misfolded ZAAT, resulting in mitochondrial ZAAT accumulation, representing a direct link between ZAAT accumulation and metabolic stress signaling, mitochondrial injury, and hepatotoxicity in AATD-mediated liver disease (Figure 8). The relevance of our findings is supported by the detection of mitochondrial ZAAT polymers in our cell culture model and in the livers of Pi*Z transgenic mice. We therefore propose that mitochondrial localization, in addition to mitochondrial ZAAT import and accumulation, are central to the pathogenesis of AATD-mediated liver disease. A better understanding of the influence of the proposed mechanisms will reveal the importance of mitochondrial dynamics for the progression of liver injury mediated by misfolded ZAAT.

## 4. Materials and Methods

### 4.1. Cell Lines and Cell Culture

In-house CRISPR/Cas9 human ZAAT-expressing Huh7.5 and wild-type Huh7.5 cells (a gift from Dr. Chen Liu) were maintained in DMEM/F12 supplemented with 10% fetal bovine serum and 100 μg/mL Primocin (InvivoGen, San Diego, CA, USA). For immunofluorescence experiments, cells were plated onto 8-well slides at a density of 20,000 cells per well. For biochemical assays, cells were plated onto six-well plates at a density of 500,000 cells per well. Cells were evaluated by Western blot, co-immunoprecipitation (co-IP), or immunofluorescence (IF).

### 4.2. Production, Maintenance, and Identification of Transgenic Mice

Transgenic animals were created using human M alpha-1 antitrypsin or Z alpha-1 antitrypsin full genome DNA (NIH gene bank Sequence ID: NC_000014.9; sequence 94374747 to 94395692), which contains both macrophage and hepatocyte enhancer promoter regions [23]. These gene sequences were inserted into pMA vectors (Invitrogen, Waltham, MA, USA) and the purified transgene DNA construct was microinjected into C57BL/6J mouse zygotes as described [59]. In this study we used 6 sex-matched (3 males and 3 females) transgenic mice at the age of 6 months old per group. Plasma samples were collected, and the mice were euthanized for liver collection. Mitochondria was extracted from fresh liver tissue. Remaining unused tissue samples were frozen in Allprotect (Qiagen, Germantown, MD, USA) and stored at −80 °C for protein and RNA analysis. The mice were maintained and all experiments performed in accordance with the National Institute of Health Guide for the Care and Use of Laboratory Animals. The Institutional Animal Care and Use Committee of the University of Florida approved all experiments conducted in this study (IACUC# 2016-3608).

### 4.3. Human AAT Transgenic Mouse Liver Tissue RNA Sequencing

The quality of the RNA-Seq sequence data was first evaluated using FastQC [60] prior to further downstream analysis. Low-quality sequences were trimmed, and poor-quality reads were removed using Trimmomatic [61]. The Star Aligner [62] was used to map high-quality paired-end reads to the mouse genome of GRCm38 [63]. Gene expression was obtained using RSEM [64]. The expected read counts and fragments per kilobase of transcript per million mapped reads (FPKM) were extracted for further analysis. The estimated read counts were used as input for edgeR [65] to perform differential expression (DE) analysis. The generalized linear model was developed to identify DE genes and the thresholds were set at FDR 0.05 and a fold change of greater than 2. Prior to the DE analysis, PCA was performed to identify outlier samples. No obvious outlier samples were found. FPKMs were used to generate a heatmap to visualize the expression pattern across samples and treatment groups.

### 4.4. Mitochondrial Extraction

Mitochondrial extraction from cultured cells was performed using the Mitochondrial Isolation Kit for Cultured Cells (Pierce Biotechnology, Rockford, IL, USA) according to the instructions. Cells were harvested via trypsinization and pelleted at 850× *g* for 5 min. The cell pellet was suspended in 800 μL of Mitochondrial Isolation Reagent A and incubated on ice for 2 min. Next, 10 μL of Mitochondrial Isolation Reagent B was added, and the cells were incubated on ice for 5 min. Then, 800 μL of Mitochondrial Isolation Reagent C was added, and the samples were centrifuged for 10 min at 700× *g*. The supernatant was transferred to a new tube and centrifuged for 15 min at 3000× *g*. The supernatant containing the mitochondria-free cytosol was transferred to a new tube and the mitochondria pellet was washed once with PBS before normalization by BCA and downstream processing.

Mitochondria were extracted from 200 mg of fresh mice liver tissue using the Mitochondrial Isolation Kit for Tissue (Pierce Biotechnology, Rockford, IL, USA). The tissue was washed twice using PBS, cut into small pieces, suspended in 800 μL of fresh PBS, and homogenized with a microhomogenizer while on ice. The tissue was centrifuged at 1000× *g* for 3 min at 4 °C and the supernatant was discarded. The pellet was suspended in 800 μL of Mitochondria Isolation Reagent A containing 4 mg/mL BSA and vortexed at medium speed for 5 s and then incubated on ice for 2 min. Next, 10 μL of Mitochondrial Isolation Reagent B was added, and the samples were incubated on ice for 5 min. Then, 800 μL of Mitochondrial Isolation Reagent C was added, and the samples were centrifuged for 10 min at 700× *g*. The supernatant was transferred to a new tube and centrifuged for 15 min at 3000× *g*. The supernatant containing the mitochondria-free cytosol was transferred to a new tube and the mitochondria pellet was washed once with PBS before normalization by BCA and downstream processing.

### 4.5. Mitochondrial DNA Isolation and Assessment

DNA was isolated from 200 μL of mouse plasma using the Qiagen DNA Mini Kit (Qiagen, Germantown, MD, USA) according to the manufacturer’s instructions. Samples were combined with 20 μL of proteinase K in microcentrifuge tubes. Then, 200 μL of Buffer AL was added and the samples were vortexed for 15 s. The samples were then incubated at 56 °C for 10 min. Next, 200 μL of ethanol was added and the samples were vortexed. The full volume was applied to a Qiagen Mini Spin Column and centrifuged for 1 min at 6000× *g*. The filtrate was discarded, and the column was washed with 500 μL of Buffer AW1 and centrifuged at 6000× *g* for 1 min. The columns were then washed with 500 μL Buffer AW2 and centrifuged for 3 min at 20,000× *g*. The columns were then placed in clean collection tubes and the DNA was eluted in 50 μL of Buffer AE.

The mitochondrial DNA content was assessed using RT-PCR. Primers for both nuclear and mitochondrial DNA were used to amplify the extracted DNA. RT-PCR samples were prepared in 20 μL reaction volumes: 10 μL SYBR master mix (Takara Biotechnology, Kusatsu, Japan), 0.8 μL each of forward and reverse primers (10 μM stocks), 6.4 μL water, and 2 μL DNA. RT-PCR was performed on the ABI-Prism 7500 System. The mtDNA copy number was calculated as follows: relative mitochondrial DNA content = 2 × 2^dCt^, where dCt = average nuclear DNA Ct − average mitochondrial DNA Ct.

### 4.6. Superoxide Measurement

Freshly extracted mitochondria (10 μg) from liver tissues was incubated with 5 μM MitoSox (ThermoFisher Scientific, Waltham, MA, USA) in HBSS at 37 °C for 30 min. Fluorescence levels were measured at ex/em 510/580 nm.

### 4.7. ROS Assay

ROS was measured using the OxiSelect Intracellular ROS Assay Kit from Cell BioLabs (San Diego, CA, USA). Whole liver tissue (20 μg) was lysed using RIPA buffer for 30 min on ice before centrifugation at 12,000× *g* for 5 min. Samples were incubated with DCFH-DA for 60 min at 37 °C and fluorescence measured at ex/em 480/530 nm.

### 4.8. Antibodies

The primary antibody for Western blot for alpha-1 antitrypsin was rabbit polyclonal from Dako (Agilent, Santa Clara, CA, USA; 1:5000). All other primary antibodies for Western blotting were from Proteintech (Rosemont, IL, USA): VDAC (1:2000), PDL-1 (1:2000), beta actin (1:5000); Cytochrome C (1:1000); GAPDH (1:5000); HSP60 (1:5000); HtrA2 (1:2000); OPA1 (1:2000); Tim23 (1:1000); Tom20 (1:10,000); Tom40 (1:1000); and Tom70 (1:1000). The secondary antibody was goat anti-rabbit (Biorad, Hercules, CA, USA; 1:3000) for regular Western blotting and Clean Blot IP Detection Reagent for IP Western blotting (ThermoFisher Scientific, Waltham, MA, USA; 1:200).

### 4.9. Histology, Histochemistry, and Immunohistochemistry

Mouse liver tissues were fixed with 4% formaldehyde and embedded in paraffin blocks, and thin sections (4 μm) were prepared. Paraffin-embedded liver tissue sections were routinely de-paraffinized with xylene and a graded series of ethanol. Some tissue sections were stained with hematoxylin–eosin (HE) for simple morphological examination. For Oil Red O staining, unfixed liver tissues were embedded frozen in optimum cutting temperature compound (Tissue-Tek) and sections (9 μm) were prepared on glass slides. Lipid staining was performed using Oil Red O (Abcam, Cambridge, UK) according to the instructions. Images were acquired using a Keyence BZ-X700 microscope. For immunostaining on paraffin-embedded liver tissues, paraffin blocks were sliced into 5 μm sections, deparaffinized with xylene, and rehydrated with decreasing concentrations of ethanol in water. Antigen retrieval was achieved by incubation for 20 min in hot (95 °C) sodium citrate buffer (pH 6.0) and 20 min of cooling at room temperature. Endogenous peroxidases were quenched by incubation in 3% hydrogen peroxide for 20 min. Sections were washed with PBS. Primary antibodies were applied for 60 min at room temperature in a humidified chamber. After rinsing the slides in PBS, they were incubated in secondary antibody for 1 h at room temperature. After washing with PBS, the slides were incubated with Vectastain ABC (Vector Laboratories, San Francisco, CA, USA) for 30 min. After washing with PBS, color development was achieved by applying diaminobenzidine tetrahydrochloride (DAB) (Vector Laboratories) for 2–5 min. Images were acquired using a Keyence BZ-X700 microscope.

### 4.10. Cholesterol Quantitation of Mouse Liver Tissues

Liver tissues cryopreserved in Allprotect from 12-month-old mice (*n* = 6) were extracted in 200 μL of chloroform:isopropanol:IGEPAL CA-630 (7:11:0.1) using a microhomogenizer. Samples were centrifuged for 10 min at 13,000× *g* to remove insoluble material. The organic phase was transferred to a new tube and allowed to air dry to remove chloroform. The samples were placed under a vacuum for 30 min to remove residual solvent. Dried lipids were suspended in Cholesterol Assay Buffer and measured using the Cholesterol Quantification Kit (Millipore Sigma, St. Louis, MO, USA). Reaction mixes were prepared according to the instructions using a probe, enzyme mix, and esterase and incubated for 60 min in the dark at 37 °C, and absorbance was measured at 570 nm.

### 4.11. Triglyceride Quantitation of Mouse Liver Tissues

Triglycerides were measured in serum and liver tissue homogenates (*n* = 6) using a triglyceride colorimetric assay kit (Cayman Chemical, Ann Arbor, MI, USA) according to the instructions. Tissue was weighed and homogenized in 2 mL of NP40 Substitute Assay Reagent and centrifuged at 10,000× *g* for 10 min at 4 °C. The supernatant was transferred to a new tube. Either 10 μL of tissue homogenate or 10 μL of serum were combined with 150 μL of the enzyme mixture. After 10 min incubation at room temperature, absorbance was read at 530 nm.

### 4.12. Mitochondrial SOD-2 Activity Assay

Mouse liver tissues (*n* = 6) were used to measure SOD-2 activity by superoxide dismutase assay kit (Cayman Chemical, Ann Arbor, MI, USA) according to the instructions. Liver tissues were centrifuged to obtain a supernatant containing cytosolic SOD and a pellet containing mitochondrial SOD. The mitochondrial pellet was homogenized in cold buffer (20 mM HEPES, pH 7.2, 1 mM EGTA, 210 mM mannitol, 70 mM sucrose) and potassium cyanide was added to the final concentration of 1 mM to inhibit Cu/Zn-SOD and extracellular SOD, resulting in the detection of only Mn-SOD activity.

### 4.13. Mitochondrial GSH Measurement

Mito-RealThiol (KeraFAST, Boston, MA, USA) was used to measure mitochondrial glutathione levels. A MitoRT probe for staining was prepared at 1 µM. A total of 2 µg of freshly isolated mitochondria was added to 100 µL of probe at 37 °C for 30 min. Relative GSH levels are reported as ratio of absorbance at 405 nm/488 nm.

### 4.14. Measurement of Mitochondrial Respiration

The oxygen consumption rate (OCR) and extracellular acidification rate of human ZAAT-expressing Huh7.5 and wild-type Huh7.5 cells were measured using a Seahorse XF96 extracellular flux analyzer (Seahorse Bioscience, Billerica, MA, USA). In brief, hepatocytes were plated on a Seahorse XF 96-well plate at a density of 2.0 × 10^4^ per well to achieve 80–90% confluency at the time of assay. Following the overnight attachment of cells, the medium was replaced with Seahorse XF medium, and the manufacturer’s protocol for the Mito Stress Kit was followed (Seahorse Bioscience). In this analysis, sequential injections of 1 μM oligomycin, 1 μM FCCP, and 0.5 μM rotenone/antimycin A were added to the cells to define the basal OCR, ATP-linked OCR, proton leak, maximal respiratory capacity, reserve respiratory capacity, and non-mitochondrial oxygen consumption. Results for mitochondrial respiration were normalized to total protein content.

### 4.15. Mitochondrial Membrane Integrity Measurement

A 20 µM JC-10 solution was prepared (AAT Bioquest, Sunnyvale, CA, USA) in Hanks Buffered Saline Solution with 20 mM HEPES and combined with 20 µg isolated mitochondria for 20 min before reading the fluorescence intensity at ex/em 490/525 nm and 540/590 nm. A MitoPT TMRM Assay from ImmunoChemistry Technologies (Bloomington, MN, USA) was performed on 20 µg of isolated mitochondria. Isolated mitochondria were incubated with TMRM reagent for 15 min at 37 °C. Samples were analyzed using a fluorescent plate reader at ex/em 540/574 nm. Samples treated with 50 µM FCCP were used as a positive control.

### 4.16. Lipid Droplet Staining and Quantification

To quantify lipid droplets, hepatocytes were fixed in 4% paraformaldehyde in PBS for 20 min followed by incubation with HSC LipidTOX red neutral lipid stain diluted in PBS for 30 min. Images were taken using a Keyence BZ-X700 fluorescence microscope.

### 4.17. Immunoblotting and Co-Immunoprecipitation

Samples (5 μg) were separated on 4–20% SDS polyacrylamide gels and transferred to nitrocellulose membranes (Bio-Rad, Hercules, CA, USA). Membranes were blocked for 1 h at room temperature and probed with antibodies. HRP-conjugated goat anti-mouse or anti-rabbit IgG antibodies (Bio-Rad) were used as secondary antibodies. To examine the interaction of AAT with mitochondrial proteins, co-IP reactions were performed. Samples were washed with cold PBS and lysed in 0.5 mL IP buffer. Approximately 1 µg of AAT antibody was added to 45 µL of protein A Dynabeads. After 10 min incubation at room temperature, the beads were washed for 3 minutes (0.1% Triton X-100 in PBS). Lysates (200 μg of total cell lysate or 100 μg mouse mitochondria) were incubated with anti-AAT-conjugated beads overnight at 4 °C. The beads were then washed, suspended in gel loading buffer, and evaluated by Western blot. Signals were developed with ChemiDoc^TM^Touch imaging system (Bio-Rad).

### 4.18. Immunostaining and Immunofluorescence Microscopy

MAAT- and ZAAT-expressing Huh7.5 cells were grown on glass coverslips. At 48 h post-seeding, the cells were fixed with 4% paraformaldehyde in PBS for 20 min and then washed. The cells were incubated for 1 h with blocking buffer (1X PBS, 5% goat serum, 0.3% Triton X-100) at room temperature, followed by overnight incubation at 4 °C with primary antibodies (1:400). The cells were washed and incubated for 1 h with secondary antibodies (Alexa Fluor 488 goat anti-mouse IgG and Alexa Fluor 594 goat anti-rabbit IgG). The coverslips were mounted and sealed. Images were collected using a Keyence BZ-X700 fluorescence microscope. The samples were scanned with a 0.1-mm step. The images were processed for brightness and contrast and filtered for noise following good practices as outlined by Rossner and Yamada.

### 4.19. Statistical Analysis

Results are expressed as mean ± S.E. Statistical analysis were performed using Prism 7 (GraphPad Software) by Student’s *t*-test or Mann–Whitney U test. Values of *p* < 0.05 were considered statistically significant.

## Data Availability

All data generated or analyzed during this study are included in this published article and its supplementary information files.

## Supplementary Materials

The following are available online at https://www.mdpi.com/article/10.3390/ijms222413255/s1.
