# Supplementary material for: The Mechanism of Mitochondrial Injury in Alpha-1 Antitrypsin Deficiency Mediated Liver Disease
Source: Int J Mol Sci. 2021 Dec 9;22(24):13255. doi: 10.3390/ijms222413255 (PMC8704552; doi:10.3390/ijms222413255)
Supplement: Supplementary file 1 [file ijms-22-13255-s001.zip › ijms-1464973-supplementary.pdf]

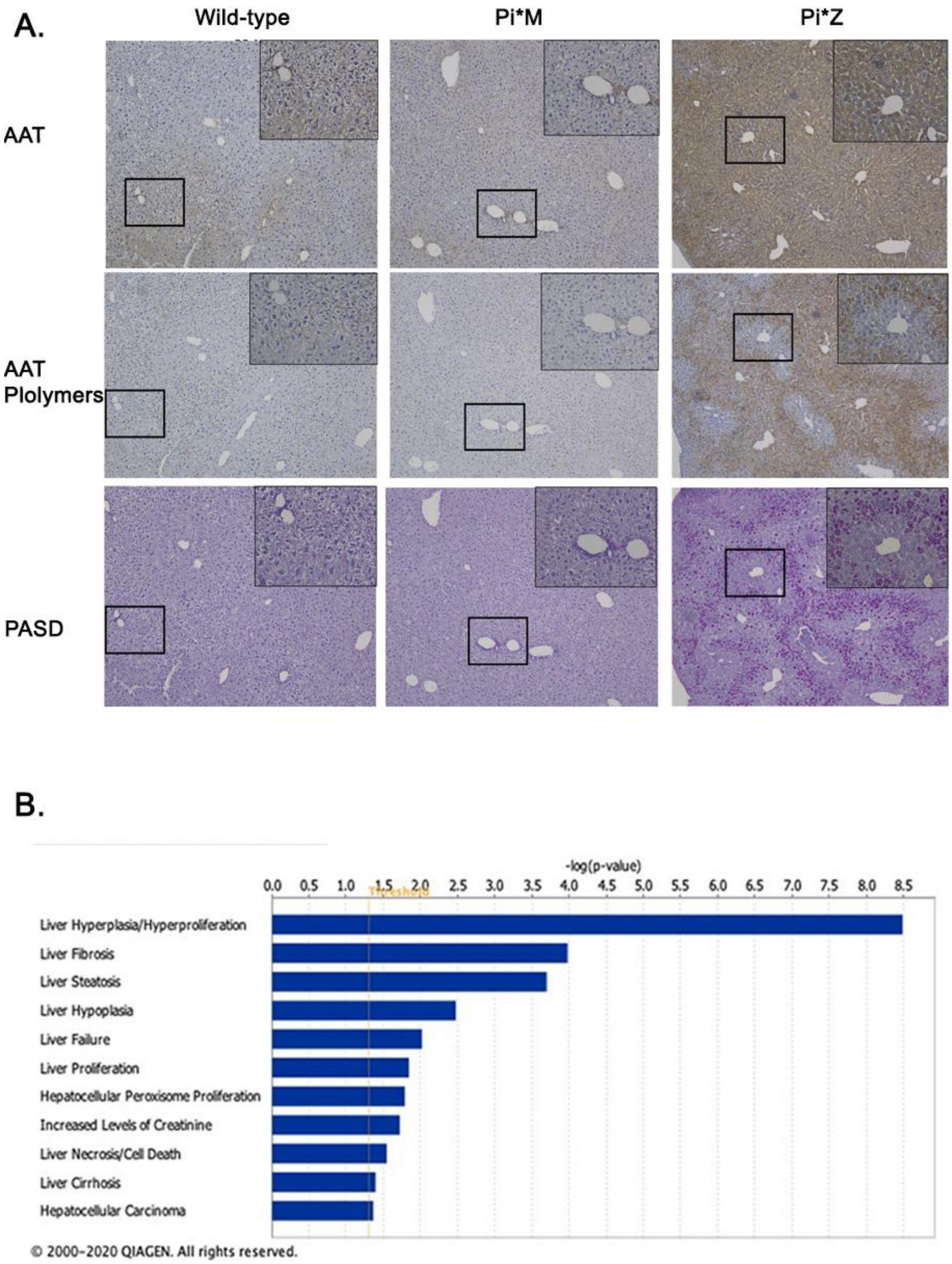

Supplementary Figure S1: AATD-mediated liver disease in Pi*Z transgenic mice. (A) Expression of human AAT in the transgenic mouse liver tissues (top row), and polymeric hAAT using rabbit 2C1 antibody against ZAAT polymers (middle row) and PASD globules (bottom row). (B). Functional repertoire of proteins which are differentially upregulated in Pi*Z liver tissues using label-free mass spectrometry using IPA (Ingenuity Pathway Analysis) software.

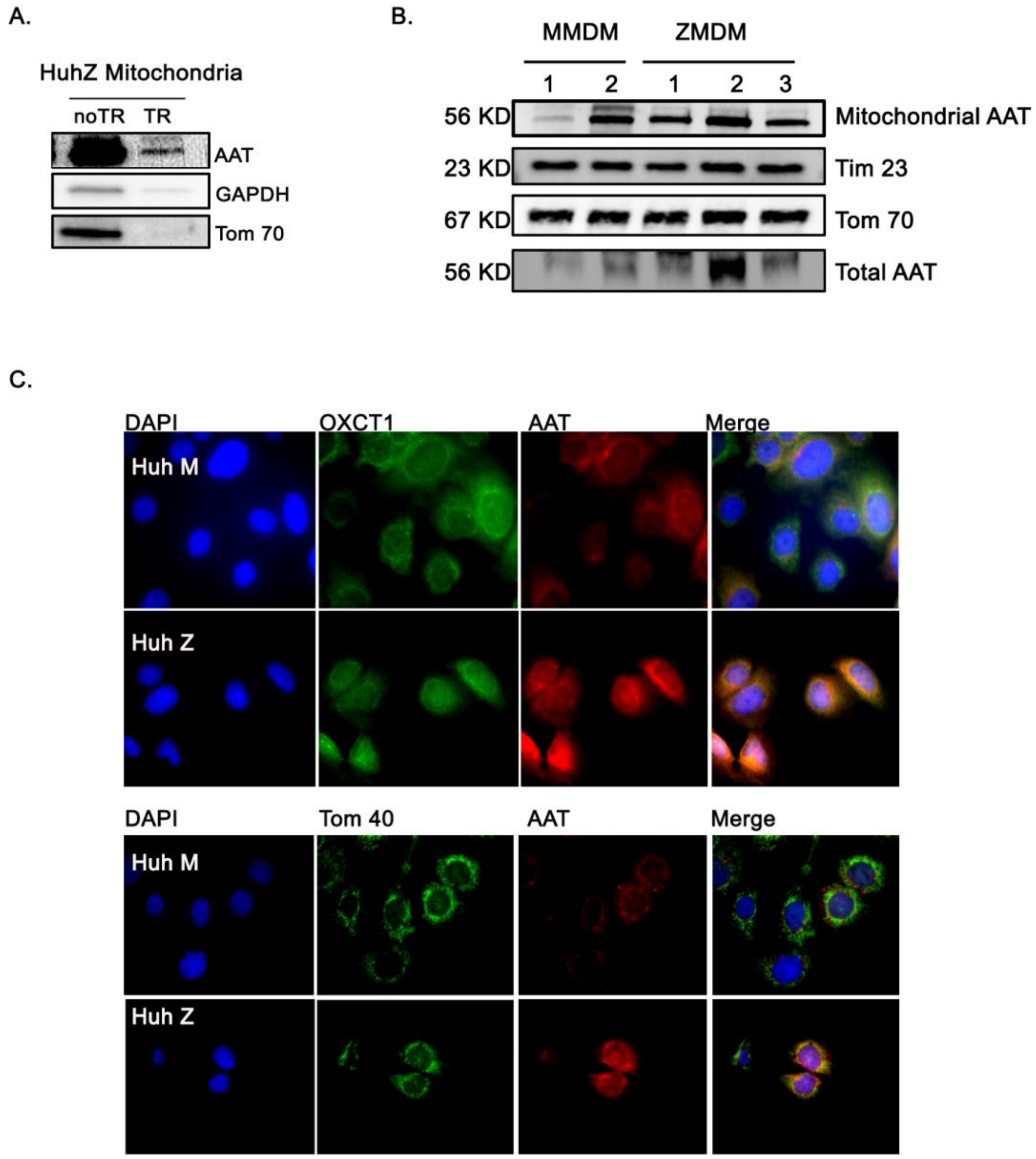

Supplementary Figure S2: ZAAT protein association with hepatic mitochondria in AATD disease model. (A) Sequential trypsin and proteinase K treatments (TR) to eliminate the substantial amount of ZAAT attached to the outside of mitochondria shown using western blot analysis. Tom70 and GAPDH have been used as mitochondrial marker and loading control, respectively. (B) Mitochondrial AAT levels in MAAT monocyte derived macrophages (MMDM) and ZAAT monocyte derived macrophages (ZMDM) (C) Double-immunofluorescence assay showing colocalization of AAT with OXCT1 and Tom40 in MAAT and ZAAT expressing Huh7.5 cells (100x).

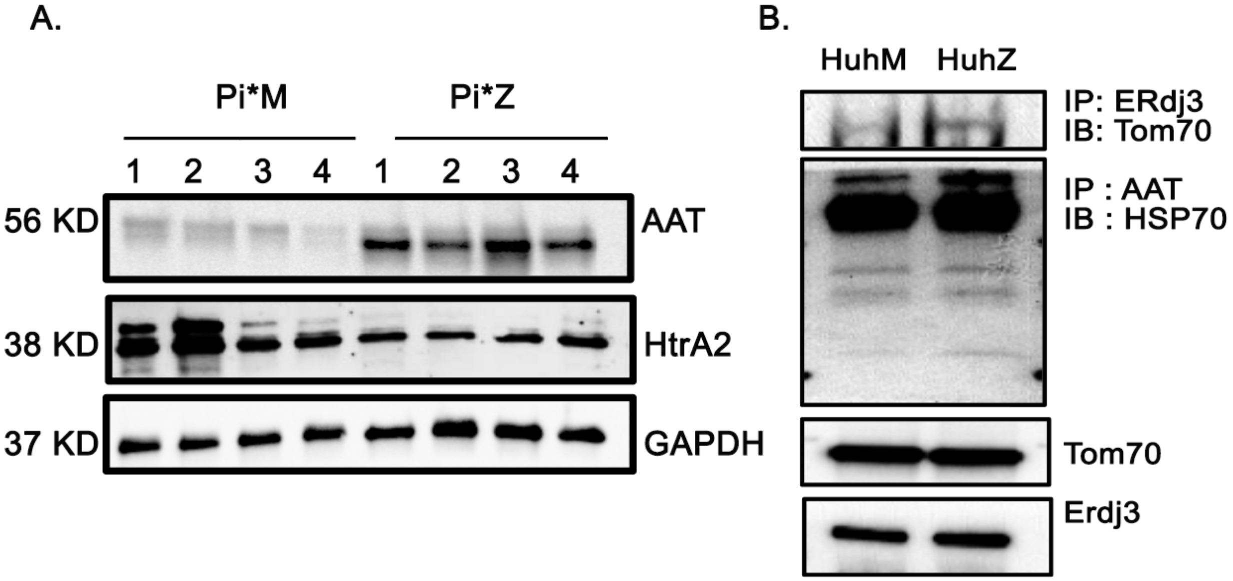

Supplementary Figure S3: Down regulation of HtrA2 in the liver of transgenic mice. (A) Western blot analysis of total AAT and HtrA2 levels from the liver of Pi*M and Pi*Z transgenic mice. GAPDH has been loaded as loading control. (B) Co-immunoprecipitation analysis indicating the interaction of ERdj3 with Tom70 in MAAT and ZAAT expressing Huh7.5 cells and cytoplasmic ZAAT accompanied by Hsp70 in ZAAT expressing Huh7.5 cells.
